# Fibroblast-directed PET imaging reveals a case of retroperitoneal fibrosis after immunotherapy for metastatic urothelial cancer of the upper tract

**DOI:** 10.1016/j.eucr.2025.103105

**Published:** 2025-06-16

**Authors:** L. Gallardo Zamora, F.C. von Rundstedt, C. Wach, W.P. Fendler, I. Maric, K. Herrmann, J.T. Siveke, P.F.-Y. Cheung, V. Grünwald

**Affiliations:** a Universitätsklinikum Wuppertal, Wuppertal, Germany; b Universitätsklinikum Essen, Essen, Germany

**Keywords:** Urothelial carcinoma, Upper urinary tract, Retroperitoneal fibrosis, Immune-related adverse event, Pembrolizumab, ^68^Ga FAPI-46 PET/CT

## Abstract

Urothelial carcinomas of the upper urinary tract (UTUC) are relatively rare and challenging to treat. We present a case report of a 55-year-old male patient with high-grade renal UTUC who received adjuvant pembrolizumab immunotherapy. The patient developed retroperitoneal fibrosis as an immune-related adverse event (irAE) following treatment.

To better understand the extent of fibrosis, we utilized ^68^Ga FAPI-46 PET/CT imaging, a radioligand that binds to fibroblast activation protein alpha expressed on activated fibroblasts. The imaging pattern correlated with histopathological findings of fibrotic tissue.

This case highlights the limited experience with rare irAEs due to checkpoint inhibitors, such as retroperitoneal fibrosis.

## Highlights

• Rare Case of Retroperitoneal Fibrosis as an irAE following pembrolizumab for metastatic UTUC.
• Only two prior cases of RPF as an irAE have been documented, making this report a significant addition to current literature.
• Detailed Clinical Case of a male with high grade UTUC, ineligible for cisplatin, who developed RPF following pembrolizumab.
• Diagnosis was confirmed using histopathology and 68Ga FAPI-46 PET/CT, highlighting the role of advanced imaging techniques.
• The report emphasizes the need for additional research for RPF in the context of irAE, biomarkers and treatment strategies.

## 1 Introduction

Urothelial carcinomas of the upper urinary tract (UTUC) represent 5–10 % of urothelial cancers, and the standard of care consists of surgery and perioperative systemic therapy, when indicated.^1^ In some cases of locally advanced UTUC, adjuvant platin-based chemotherapy and checkpoint inhibitors are considered as standard of care. The PD-1 inhibitor pembrolizumab has been approved in patients with PD-L1 positive advanced or metastatic urothelial carcinoma ineligible for cisplatin-based first-line chemotherapy.^2^^,^^3^ PD-1 inhibitors can cause multiple immune-related adverse events (irAEs).^4^ Retroperitoneal fibrosis as an irAE following anti-PD-1 immunotherapy is rare event^5^^,^^6^^,^^7^**,** but its diagnosis poses a clinical challenge.

## 2 Case presentation

A 55-year-old male patient presented with back pain. An abdominal CT scan demonstrated a left renal mass and multiple enlarged paraaortic lymph nodes. The primary chest CT was unremarkable. Ureterorenoscopy biopsy revealed high-grade urothelial carcinoma, and primary surgery was performed.^1^

The patient underwent left robotic nephroureterectomy with template-based hilar and para-aortic lymphadenectomy. The final histopathological examination revealed pT3 high-grade renal UTUC with four positive lymph nodes and negative surgical margins. Immunochemistry showed a combined positive score (CPS) of 100. Adjuvant cisplatin-based chemotherapy was not offered because of renal insufficiency (GFR 43 ml/min/L) and pre-existing hearing impairment.^1^^,^^8^

Our interdisciplinary tumor board recommends adjuvant immunotherapy with pembrolizumab.^3^^,^^4^ Treatment was initiated with 200 mg pembrolizumab every three weeks.^9^

The patient completed the 15 weeks of treatment. Routine computed tomography (CT) revealed a new retroperitoneal mass extending from the cranial pole of the right renal artery downward to the aortic bifurcation and into the root of the small bowel mesentery. An exploratory laparotomy revealed an unresectable fibrotic mass encasing the aorta and infiltrating the mesenteric root.

An excisional biopsy was performed. Pathological examination ruled out malignancy and indicated extensive fibrotic tissue. Clinical progression occurred within four weeks, and right-sided hydronephrosis required stenting of the solitary kidney. The patient was started on corticosteroid therapy for retroperitoneal fibrosis at an initial dose of 1 mg/kg.^10^

Nuclear imaging with abdominal PET/CT ^18^F-FDG showed increased metabolic activity in retroperitoneal lesions. For clarification, an additional ^68^Ga FAPI-46 PET/CT scan was performed. ^68^Ga FAPI-46 is a radioligand that binds with high affinity to the fibroblast activation protein alpha (FAP) on the cell surface of activated fibroblasts, thereby enabling the imaging of fibrosis.^11^

**Fig. 1:**
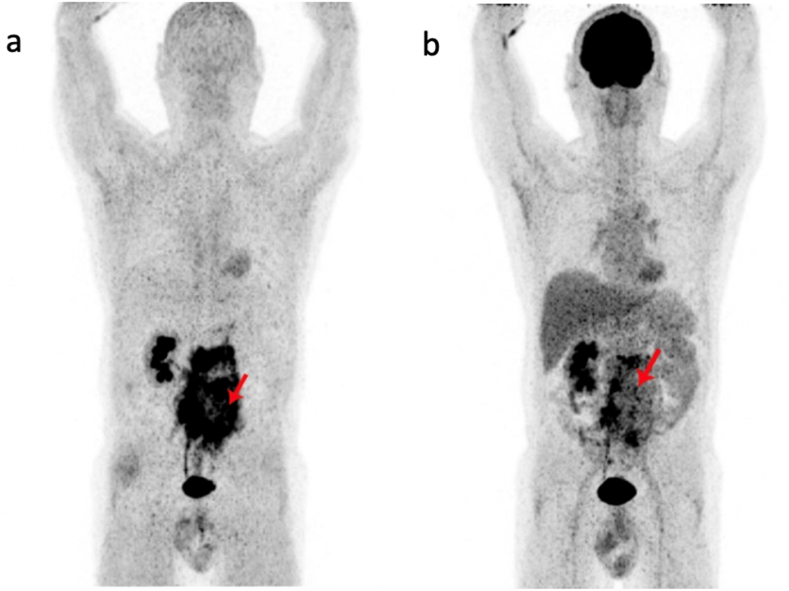
[68Ga]FAPI PET/CT (a) and 18FFDG PET/CT (b) images of restaging for urothelial carcinoma of the renal pelvis undergoing immunotherapy with pembrolizumab. Arrows indicate the retroperitoneal mass with high FAP-expression (left row) and intense, inhomogenous FDG-avidity (right row).

**Fig. 2:**
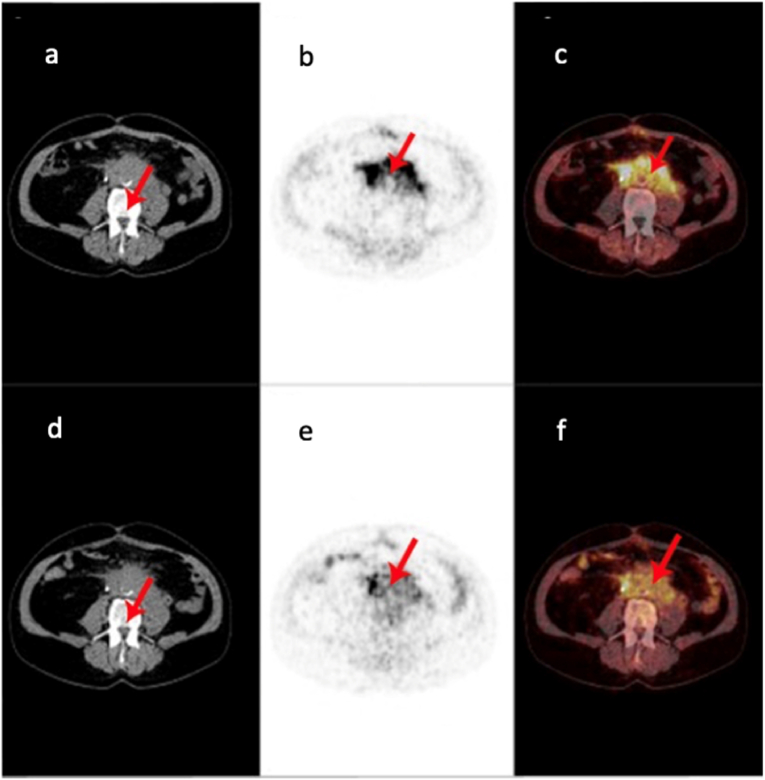
[68Ga]FAPI PET/CT (a–c) and 18FFDG PET/CT (d–f) images of restaging for urothelial carcinoma of the renal pelvis undergoing immunotherapy with pembrolizumab. Arrows indicate the retroperitoneal mass with high FAP-expression (upper row) and intense, inhomogenous FDG-avidity (lower row).

By analyzing ^68^Ga FAPI-46 PET/CT, we observed increased uptake within the retroperitoneal mass, but a more diffuse pattern compared to ^18^F-FDG PET CT, as illustrated in Fig. 1, Fig. 2.

**Fig. 3:**
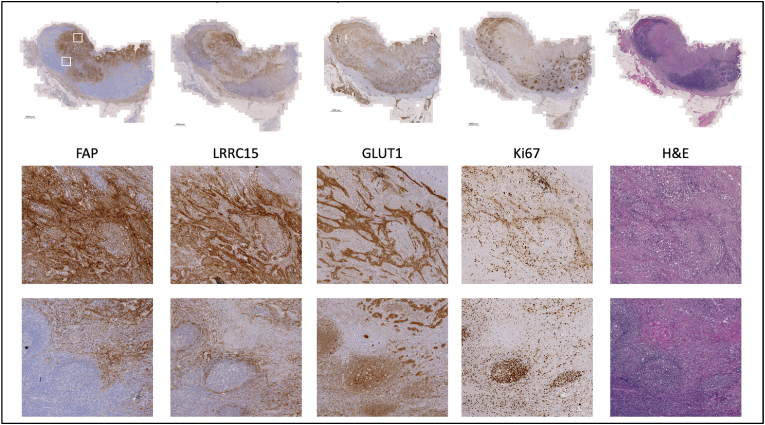
FAP staining for the lymph node resected at the exploratory laparotomy. FAP staining shows a strong expression level.

To correlate the findings of the ^68^Ga PET/CT FAPI-46 with histopathological findings, we performed immunohistochemical staining of FAP in the retroperitoneal tissue obtained from the open laparotomy. As presented in Fig. 3, FAP immunostaining of the surgical biopsy was consistent with the findings of fibrotic tissue in PET scans, demonstrating overall intermediate expression levels in multiple biopsy locations.

**Fig. 4:**
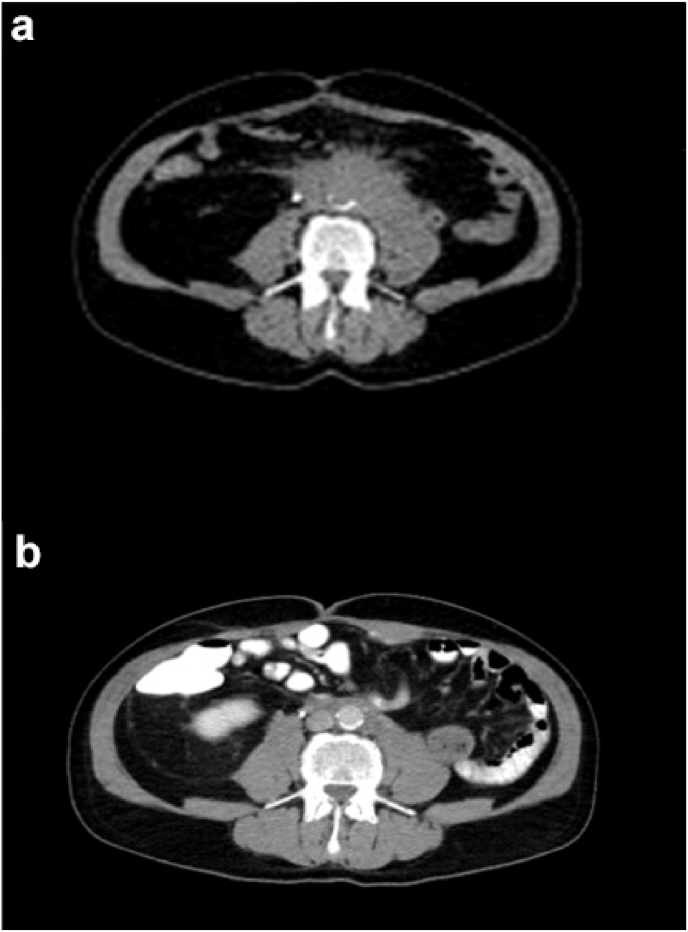
CT images before (a) and after (b) introducing steroid treatment with significant decrease in size of the retroperitoneal mass.

Secondary retroperitoneal fibrosis was diagnosed as an immune-related adverse event, and steroid treatment was continued. After six months of steroid treatment, the retroperitoneal fibrosis **had regressed**, and the ureteral stent was successfully removed, as evidenced in Fig. 4 by the significant reduction in mass size on CT imaging before and after treatment.

The oral prednisolone therapy was tapered to a maintenance dose of 5 mg/day. Twelve months after the initiation of corticosteroid therapy, a follow-up CT scan revealed a new mass around the psoas muscle, triggering a new ^18^F-FDG PET CT scan, which showed a new, metabolically avid lesion. CT-guided biopsy revealed fibrotic tissue without the presence of urothelial carcinoma. Progression of retroperitoneal fibrosis required a rechallenge with corticosteroid steroid therapy at an initial dose of 1 mg/kg.

Two and a half years after the diagnosis of UTUC, the patient developed disseminated bipulmonary, mediastinal lymph node, and bone metastases to the thoracic spine and rib. Chemotherapy with gemcitabine and carboplatin was initiated, but had to be discontinued because of toxicity. The patient died three years after the initial diagnosis.

## 3 Discussion

This case emphasizes the limited experience with checkpoint inhibitor drugs in locally advanced and metastatic urothelial cancer.

The treatment of cancers with programmed cell death protein 1 (PD-1/PD-L1) pathway inhibitors can lead to a wide range of immune-related adverse events (irAEs).^5^

The KEYKNOTE-052 trial investigated the use of pembrolizumab in platin-ineligible patients with locally advanced and metastatic urothelial carcinoma. Secondary retroperitoneal fibrosis was not reported as a treatment-related adverse event, also not for UTUC.^3^ **Only a few case reports have described retroperitoneal fibrosis following immunotherapy. In all reported cases, patients were treated empirically with corticosteroids, either based on clinical suspicion**^7^ **or after histological confirmation by biopsy**^12^**, and all showed a favorable response with regression of the fibrotic tissue. The corticosteroid dose and duration of therapy varied between cases, but treatment was consistently administered over a period of several months.**

Retroperitoneal fibrosis is an immune-mediated disease characterized by the development of inflammation and fibrosis in the soft tissues of the retroperitoneum and other abdominal organs. Fibrosis mostly exhibits symmetrical growth patterns around the aorta beneath the renal vessels, down to the division of the aorta and common iliac arteries. It typically involves the ureter and commonly results in hydronephrosis and renal failure.

Multiple drugs, such as ergot alkaloids or TNF alpha-blockers, have been associated with retroperitoneal fibrosis.^6^^,^^7^ Steroid therapy is the gold-standard treatment for retroperitoneal fibrosis^13^ **with the exception of malignant retroperitoneal fibrosis. It accounts for approximately 10 % of all retroperitoneal fibrosis cases and is linked to a significantly worse prognosis than idiopathic forms. Management primarily targets the underlying malignancy, as corticosteroids have not shown proven efficacy in malignant retroperitoneal fibrosis, except in cases associated with carcinoid tumors**^14^**.**

Differentiating rare irAEs from cancer progression is a persistent clinical challenge, further complicated by the uncertainty of whether observed changes reflect true immune-related responses or the natural course of tumor evolution; an ambiguity that limits the interpretation of therapeutic outcomes, especially in complex tumor presentations.

Reliable imaging modalities for differentiating between cancer and other diseases remain scarce.

PET/CT ^18^F-FDG has limitations in differentiating between cancer and inflammatory metabolic activities.^8^

^68^Ga PET/CT FAPI-46 has been increasingly used over the past years in the diagnosis and staging of tumors and fibrotic entities that correlate with the high activity of fibroblasts. **It has shown strong potential as an oncological theranostic tool in several studies**^15^^,^^16^**, due to its markedly higher uptake in tumor tissue and low background signal, enabling precise lesion visualization. Its application may be particularly valuable for monitoring treatment response, especially following immune checkpoint inhibition, where conventional imaging with PET/CT ^18^F-FDG can be misleading due to pseudoprogression.**

In this clinical case, ^68^Ga PET/CT FAPI-46 proved useful in characterizing the fibrotic process. Although ^68^Ga PET/CT FAPI-46 has not yet been specifically evaluated in upper tract urothelial carcinoma, its utility in assessing urological cancers has been explored.^11^ To better define the diagnostic performance of FAP-targeted imaging in the setting of immune-related adverse events, further prospective studies with larger patient cohorts are necessary.

Targeted molecular imaging may assist clinicians in monitoring the immune responses to targeted cancer therapies. Ideally, this should be considered in combination with liquid biopsy. Serial liquid biopsy with a broad-panel next-generation sequencing assay that sequences to a very high depth of coverage would be an excellent way to follow these patients, particularly if there is a baseline genomic profile for their tumor.

## 4 Conclusion

Retroperitoneal fibrosis after immunotherapy is a rare event but poses a significant challenge as the oncologist may be tempted to assume cancer progression when long-term steroids should be the treatment of choice.

A multidisciplinary approach, including urologists, oncologists, radiologists, and expert pathologists, is crucial for managing these cases. Additionally, targeted molecular imaging and may aid in monitoring immune responses and guiding treatment decisions in patients receiving targeted cancer therapies.

## Declaration of interests Laura Gallardo Zamora for submitting the case report

☒ The authors declare that they have no known competing financial interests or personal relationships that could have appeared to influence the work reported in this paper.

## CRediT authorship contribution statement

**L. Gallardo Zamora:** Writing – review & editing, Writing – original draft, Conceptualization. **F.C. von Rundstedt:** Writing – review & editing, Methodology, Conceptualization. **C. Wach:** Data curation, Conceptualization. **W.P. Fendler:** Writing – review & editing, Supervision, Methodology, Investigation. **I. Maric:** Resources. **K. Herrmann:** Resources. **J.T. Siveke:** Writing – review & editing, Resources. **P.F.-Y. Cheung:** Resources. **V. Grünwald:** Writing – review & editing, Supervision, Methodology, Conceptualization.

## Ethics approval and consent to participate

This clinical case was approved by the ethical committee of the Universitätsklinikum Wuppertal, which is comprised by oncologists, urologists, radiologists, pathologists, radiotherapists.

## Consent for publication

Consent for publication was obtained and signed by the patient. We confirm, that the patient gave written informed consent for his clinical details and images to be published in this case report.

## Availability of data and materials

The data supporting the findings of this study were sourced from PubMed and Cochrane Reviews.

## Funding

This research received no external funding and was supported by the Department of Urology of the Universitätsklinikum Wuppertal.

A declaration of interests has been made using the form provided by *Urology Case Reports* and will be submitted as a separate file.

## Declaration of competing interests

All authors declare that they have both financial and non-financial competing interests.
